# Quantification of [^99^Tc]TcO_4_^-^ in urine by means of anion-exchange chromatography–aerosol desolvation nebulization–inductively coupled plasma–mass spectrometry

**DOI:** 10.1007/s00216-024-05149-4

**Published:** 2024-01-30

**Authors:** Maximilian Horstmann, C. Derrick Quarles, Steffen Happel, Michael Sperling, Andreas Faust, Kambiz Rahbar, David Clases, Uwe Karst

**Affiliations:** 1 Institute of Inorganic and Analytical Chemistry, University of Münster, Münster, Germany; 2 Elemental Scientific, Inc., Omaha, NE USA; 3 TrisKem International SAS, Bruz, France; 4 European Virtual Institute for Speciation Analysis (EVISA), Münster, Germany; 5 European Institute for Molecular Imaging (EIMI), Münster, Germany; 6 Department of Nuclear Medicine, University Hospital Münster, Münster, Germany; 7 West German Cancer Center, Münster, Germany; 8 Institute of Chemistry, University of Graz, Graz, Austria

**Keywords:** Pertechnetate, Elemental speciation, Hyphenated ICP-MS techniques, On-line calibration, Trace analysis

## Abstract

To sensitively determine ^99^Tc, a new method for internal quantification of its most common and stable species, [^99^Tc]Tc${\text{O}}_{4}^{-}$, was developed. Anion-exchange chromatography (IC) was coupled to inductively coupled plasma–mass spectrometry (ICP-MS) and equipped with an aerosol desolvation system to provide enhanced detection power. Due to a lack of commercial Tc standards, an isotope dilution-like approach using a Ru spike and called isobaric dilution analysis (IBDA) was used for internal quantification of ^99^Tc. This approach required knowledge of the sensitivities of ^99^Ru and ^99^Tc in ICP-MS. The latter was determined using an in-house prepared standard manufactured from decayed medical ^99m^Tc-generator eluates. This standard was cleaned and preconcentrated using extraction chromatography with TEVA resin and quantified via total reflection X-ray fluorescence (TXRF) analysis. IC coupled to ICP-MS enabled to separate, detect and quantify [^99^Tc]Tc${\text{O}}_{4}^{-}$ as most stable Tc species in complex environments, which was demonstrated in a proof of concept. We quantified this species in untreated and undiluted raw urine collected from a patient, who previously underwent scintigraphy with a ^99m^Tc-tracer, and determined a concentration of 19.6 ± 0.5 ng L^−1^. The developed method has a high utility to characterize a range of Tc-based radiopharmaceuticals, to determine concentrations, purity, and degradation products in complex samples without the need to assess activity parameters of ^99(m)^Tc.

**Graphical Abstract:**

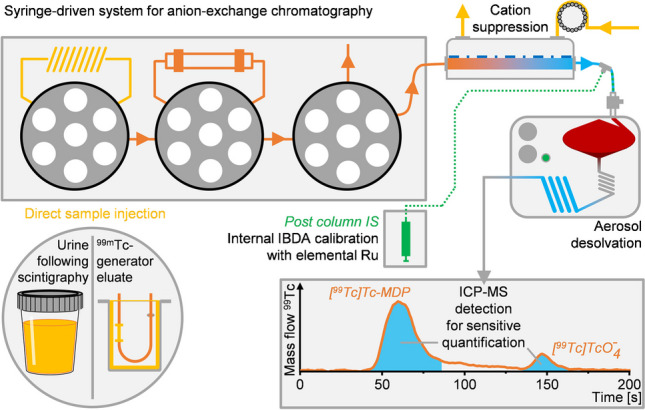

## Introduction

### Technetium in clinical applications

Until the mid-1930s, the very center of the periodic table held a blank spot, which was reserved for element 43 and, which had yet to be discovered. In 1937, Emilio Segre and Carlo Perrier were the first to isolate the element, which — referring to its synthetic origin — was named technetium. Unlike its elemental neighbors, all known isotopes of the element are unstable and have a half-life varying between 100 ns and 4.2·10^6^ years [1]. The decay of its isotope ^99m^Tc plays a major role in diagnostic medicine [2] and can be exploited for single-photon emission computed tomography (SPECT) [3]. During the examination, ^99m^Tc transitions to its ground state ^99^Tc emitting a y-quant with an energy of 140.5 keV at a yield of more than 98% and a half-life of 6 h [1, 4]. These unique characteristics, as well as its easy accessibility via mobile generators and its versatile chemistry promoted the synthesis of a vast range of pharmaceuticals and made ^99m^Tc the most important radionuclide, which is used in approx. 30 million examinations every year [4–6]. The multifaceted chemistry of ^99m^Tc enables the synthesis of specific complexes in commercial labelling kits, which can subsequently be administered to patients to target specific organs and structures in diagnostic imaging [6, 7].

**Fig. 1:**
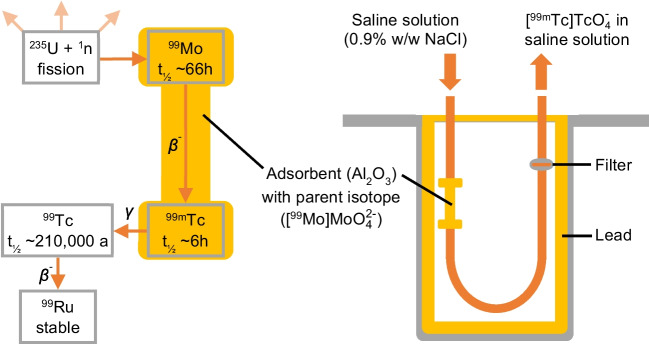
Schematic depiction of a ^99m^Tc-generator used in hospitals to produce [^99m^Tc]Tc${\text{O}}_{4}^{-}$ for the synthesis of scintigraphy tracers. The decay chain of ^99m^Tc, produced from ^235^U down to the final stable isotope ^99^Ru, is depicted on the left side [1, 9]

Inside the hospital, ^99m^Tc and its respective tracers are prepared individually for each examination using ^99m^Tc-generators (Fig. 1) [8]. These generators contain the parent radioisotope ^99^Mo, which is immobilized as [^99^Mo]Mo${\text{O}}_{4}^{2-}$ on an Al_2_O_3_ column and can be distributed to the medical facilities to obtain ^99m^Tc on-site. On the column, ^99^Mo decays with a half-life of 66 h and almost exclusively forms the desired radionuclide ^99m^Tc in its most stable species [^99m^Tc]Tc${\text{O}}_{4}^{-}$ [1]. Still adhered to the column, [^99m^Tc]Tc${\text{O}}_{4}^{-}$ can be selectively eluted with physiological saline solution as the doubly charged species of the parent isotope [^99^Mo]Mo${\text{O}}_{4}^{2-}$ shows stronger affinity to the carrier material [3, 9]. The saline solution containing active ^99m^Tc can immediately be modified to obtain the tracer and administered with no further clean-up [7]. As such, radiopharmaceutical formulations may contain impurities and contaminations, which however are little explored [10].

### Quantification of Tc

Especially in view of the half-life of ^99m^Tc (Fig. 1), time plays a critical role in the conduction of scintigraphy examinations [10]. For a typical SPECT scan, it often only takes a few hours between the generation of the radionuclide on a ^99m^Tc-generator, the synthesis of the organ-specific tracer, the actual scan, and tracer excretion [5, 11]. During these time-sensitive steps, analysis of the tracer is only conducted by determining the ^99m^Tc activity. As long as the solution shows sufficient γ-activity, it can consecutively be labelled and injected. Commonly administered tracers include [^99m^Tc]Tc-DTPA ([^99m^Tc]Tc-pentetic acid; brain, kidney, or lung scintigraphy), [^99m^Tc]Tc-sestamibi ([^99m^Tc]Tc-Hexakismethoxyisobutylisonitrile; cardiac muscle and parathyroid gland scintigraphy), or [^99m^Tc]Tc-MDP (^99m^Tc-methylene diphosphonate, bone scintigraphy) [7, 12, 13]. Prior to examination, the total amount of administered ^99^Tc is unknown, which may present a pitfall when studying labelling efficiency or when examining the fate of tracers [14, 15]. To reach the required sensitivity to consider total Tc levels, mass spectrometric tools are applicable [16]. Especially elemental mass spectrometry, i.e., inductively coupled plasma–mass spectrometry (ICP-MS), provides high sensitivity and unique quantification strategies which may overcome current analytical challenges []. Compared to the detection of β-emission, ICP-MS can achieve higher sensitivity and sample throughput, as neither analyte preconcentration nor tedious separation from other radioactive matrix compounds prior to analysis is required [17]. Furthermore, it pairs high matrix tolerance with the possibility to couple different separation methods and thus combines low detection limits in the sub-ng L^−1^ range with the opportunity to consider element species [15, 17]. Specifically when investigating the fate of ^99^Tc in environmental samples from areas with nuclear contamination, ICP-MS has become the method of choice [18–21]. One example for its utility to carry out Tc speciation analysis was demonstrated in our recent study, where we used LC-ICP-MS in conjunction with molecular mass spectrometry to study impurities in radiopharmaceuticals [10].

One of the major challenges for the determination of ^99^Tc by means of ICP-MS is the lack of available element standards. One way to bypass this issue includes a calibration strategy called isobaric dilution analysis (IBDA), which uses a Ru spike exhibiting an isobaric isotope and records the isotope ratio (99/101) for calibration [10, 22]. Similar to isotope dilution analysis (IDA), this approach can be applied either off-line or on-line by continuously spiking a Ru solution after a chromatographic column. The latter provides opportunities to even calibrate unknown Tc species [10].

In this work, an anion-exchange chromatography (IC) method was developed and coupled to ICP-MS enabling on-column preconcentration of [^99^Tc]pertechnetate ([^99^Tc]Tc${\text{O}}_{4}^{-}$) and other ^99^Tc-containing species, which were subsequently quantified via IBDA. This promoted lower detection limits while tolerating complex matrix compositions. Using an automated inline sample preparation and chromatography system enabled accurate flow control of both eluent and post-column spike, thereby increasing the overall accuracy of IBDA compared to previous approaches. By employing aerosol desolvation nebulization, sensitivity could be further increased relative to conventional sample introduction strategies for ICP-MS [23]. An elemental ^99^Tc standard was prepared in-house from ^99m^Tc-generator eluates and quantified via total reflection X-ray fluorescence (TXRF) analysis. The standard allowed direct determination of the specific elemental sensitivity of ^99^Tc in ICP-MS bypassing previous approximation methods and thus, increasing accuracy and precision [22]. The newly developed method provides new possibilities to perform ultra-trace analysis of [^99^Tc]Tc${\text{O}}_{4}^{-}$ in complex matrices. In a proof of principle, this study demonstrates the developed method to determine [^99^Tc]Tc${\text{O}}_{4}^{-}$ in urine of a scintigraphy patient.

## Materials and methods

### Chemicals and consumables

Bidistilled water was obtained with an Aquatron water still purification system model A4000D (Barloworld Scientific, Nemours, France). Elemental standards of Y, Zr, Nb, Mo, Ru, Rh, Pd, Cd, and As (all 1000 mg/L) were purchased from Merck KGaA (Darmstadt, Germany). Nitric acid (65%, AnalaR NORMAPUR®, (w/v)) and sulfuric acid (95%, AnalaR NORMAPUR, (w/v)) were purchased from VWR International (Radnor, PA, USA). Ammonium hydroxide solution (25%, Analytical Reagent Grade, (w/v)) was purchased from Fluka Chemie GmbH (Buchs, Switzerland). Ammonium nitrate (p.A.) was acquired from AppliChem GmbH (Darmstadt, Germany). Polypropylene sample tubes (15 and 50 mL) were obtained from Th. Geyer (Renningen, Germany) and syringe filters (0.45 µm, 25 mm, hydrophilic PTFE) were purchased from BGB Analytik AG (Boeckten, Switzerland). Empty cartridges for extraction chromatography were purchased from Merck KGaA (Darmstadt, Germany). TEVA resin used for the extraction chromatographic purification of [^99^Tc]Tc${\text{O}}_{4}^{-}$ was provided by Triskem International SAS (Bruz, France).

### Mass spectrometric detection

All ICP-MS experiments were conducted on an Agilent 7700 ICP-MS (Agilent Technologies, Santa Clara, CA, USA) with x-lens configuration and platinum sampler and skimmer cones using MassHunter software (MassHunter 4.6, Version C.01.06). The ICP-MS was tuned daily to obtain an optimal signal-to-noise-ratio (*S*/*N*) for the *m*/*z* of 99 and 101. Typical instrument parameters were as follows: RF power, 1600 W; RF matching, 1.52 V; sample depth, 5.5 mm; first extraction lens, − 10 V; second extraction lens, − 200 V; omega bias, − 105 V; omega lens, 5.4 V; cell entrance, − 40 V; cell exit, − 56 V; deflect, 12.6 V; plate bias, − 40 V; octopole bias, − 8.5 V; octopole RF, 180 V; energy discrimination, 7.1 V. The measurements were conducted without the need for a collision gas flow as backgrounds were sufficiently low and polyatomic interferences were not expected. Detector dead time was calibrated using an elemental In standard (10 mg/L and 50 µg/L). Dwell times for the *m*/*z* ratios 99 and 101 were set at 0.4 s to decrease the relative standard deviation of the recorded signals and to improve precision for the recorded isotope ratio. To additionally detect potential interferences of Mo, the *m*/*z* of 97 was monitored with a dwell time of 0.1 s. Mass bias correction was performed using the four most abundant isotopes of Ru with the exponential approach suggested by Rodriguez Gonzalez et al. [24].

To increase sensitivity, aerosol desolvation nebulization was performed with an Apex 2 High Sensitivity Desolvating System equipped with a MicroFlow PFA ST nebulizer operated with Apex software (Version 1.0.1.2, all from Elemental Scientific, Omaha, NE, USA). The operating parameters were tuned concurrently with the instrumental parameters of the ICP-MS: nebulizer gas flow, 0.85 L/min; argon makeup gas flow, 0.311 L/min; N_2_ add-gas flow, 3.9 mL/min; spray chamber temperature, 140 °C; condenser temperature, 3 °C.

### Chromatographic separation

**Fig. 2:**
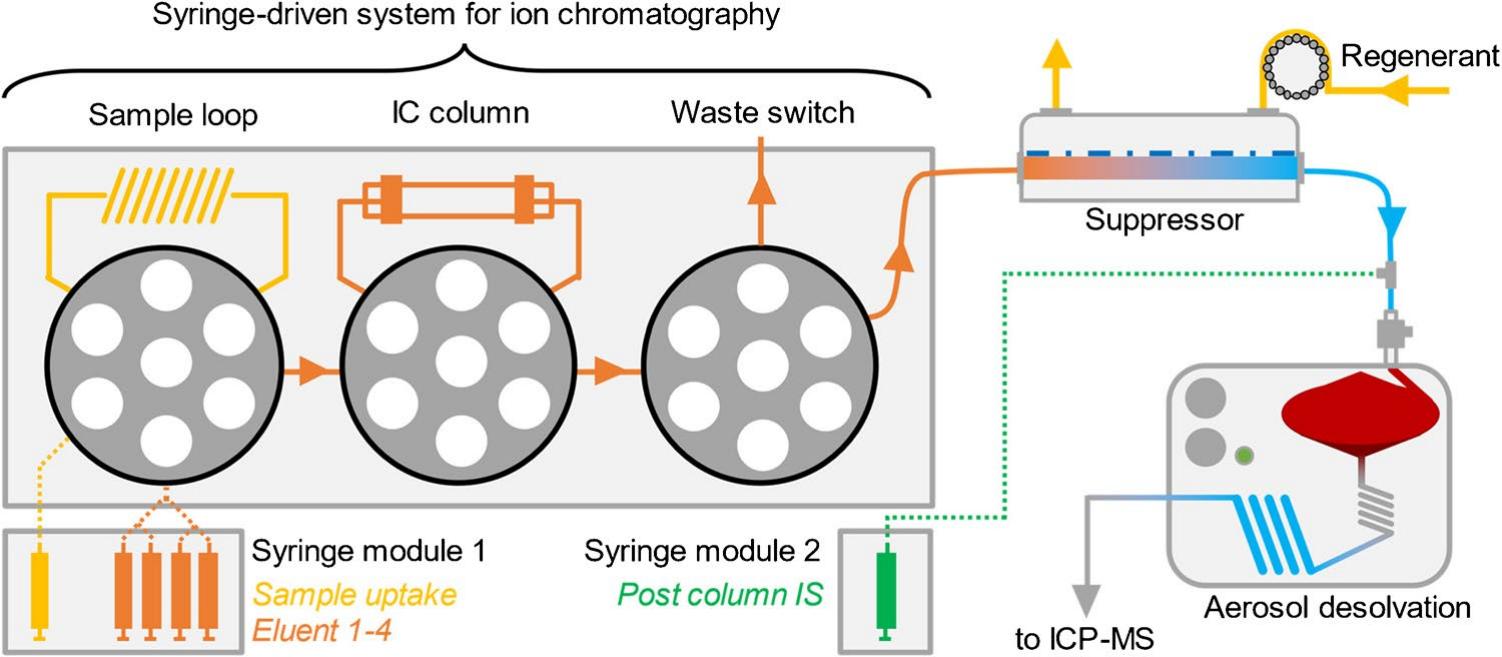
Schematic depiction of the developed setup for on-line ion chromatographic quantification of [^99^Tc]Tc${\text{O}}_{4}^{-}$ with IBDA using ion suppression and aerosol desolvation nebulization coupled to ICP-MS

The IC separation was conducted using a fully automated single platform system for total metal analysis and syringe-driven chromatography (prep*FAST* IC, Elemental Scientific, Omaha, NE, USA) that was configured to fulfill the specific needs of the described analysis (Fig. 2). Separation was achieved with an IonPac AG9-SC column (4 × 50 mm, Thermo Fisher Scientific, Waltham, MA, USA). Method development and system operation were performed using ESI SC (Version 2.9.0.496, Elemental Scientific, Omaha, NE, USA). Compared to the standard setup that allowed auto dilution of standards and samples within two separate loops, only one sample loop of 50 µL was installed as no dilution of the sample was necessary [25–27]. Instead, four chromatographic syringes were used to hold different eluents or rinsing solutions to set up a step gradient consisting of 150 mM NH_4_NO_3_ solution (eluent A, set to pH 9.2 with NH_4_OH solution) and bidistilled water (eluent B). 500 mM NH_4_OH solution (eluent C, rinsing) and 20 mM nitric acid (eluent D, rinsing) were prepared for rinsing the column as well as the chromatographic system before and after each injection. The developed three-step gradient was run at a flow rate of 650 µL/min and initially consisted of eluents A and B in a composition of 10% A and 90% B. After 40 s, the concentration was increased to 50% A until it was changed to 100% A after another 90 s. For quantification, a Ru post-column internal standard (PCIS; concentration 1 µg/kg, in 10 mM HNO_3_) was added at a flow rate of 50 µL/min through another syringe, so that the overall flow rate was set at 700 µL/min. After the chromatographic separation, the column was first rinsed with eluent C (60 s, 700 µL/min) and the chromatographic system and the loop were finally rinsed with eluent D (60 s, 700 µL/min). Column recoveries were determined by comparing flow injections of different diluted solutions of the quantified ^99^Tc standard with respective column injections by changing the position of the column valve accordingly. The examined spiked sample of ^99m^Tc-generator eluate was previously diluted by a factor of 10,000, to reduce the effects of its matrix containing 0.9% w/w NaCl and to test the method at a concentration closer to its lower detection capabilities.

The application of the aerosol desolvation system requires considerations for used buffers and especially in cases where non-volatile salts are used, crystal build-up can be a limiting factor [23]. To avoid formation of crystalline NH_4_NO_3_ within the aerosol desolvation nebulization system, a Dionex ACRS 500 chemically regenerated suppressor (4 mm ID, Thermo Fisher Scientific, Waltham, MA, USA) was installed between the chromatographic system and the sample introduction system. H_2_SO_4_ (150 mM) was chosen as a regenerant using an external peristaltic pump at a flow rate of 2.3 mL/min to suppress the ammonium cations originating from the eluent. Individual recovery of the suppressor was determined by flow injection of a diluted solution of the quantified ^99^Tc standard either flowing through or bypassing the suppressor.

### Sample handling and preparation

The urine sample was collected from an anonymous patient, who underwent a bone scintigraphy using a [^99m^Tc]Tc-MDP tracer. The study was approved by the local ethics committee (No. 2007-467-f-S and No. 2016–585-f-S, Ethikkommission der Ärztekammer Westfalen-Lippe und der Universität Münster). The sample was collected, immediately frozen at − 80 °C and left in the dedicated environment at the university hospital for a period spanning several half-lives of ^99m^Tc until no increased activity was detectable. Prior to analysis, the sample was thawed, filtered with a syringe filter (0.45 µm, 25 mm, hydrophilic PTFE) and directly analyzed with the same setup presented in the experimental section above, which was used for all presented chromatography data. This involved the injection of 50 µL raw and undiluted urine onto the IC column immediately after the sample was filtered, which represented a very low preparation and analysis effort even for samples containing a strong matrix composition.

### Generation and quantification of ^99^Tc standard

For on-line quantification of ^99^Tc using IBDA, sensitivity correction between ^99^Tc and ^99^Ru was necessary. To increase the accuracy of the sensitivity correction, an in-house elemental ^99^Tc standard was prepared from ^99m^Tc-generator eluates. The raw eluate was stored in glass bottles and left in the dedicated environment at the university hospital until no increased activity through γ-radiation was detectable. To decrease potential exposure and limit the risks, associated especially with the use of higher concentrations of the newly generated standard, all vessels as well as all unprotected instrumental setups containing higher concentrations of ^99^Tc were covered in aluminum foil, to prevent any β-radiation from penetrating. As the largest hazard of ^99^Tc originates from contamination through small particles or aerosols which can show persistent behavior in the lungs, the formation of aerosols as well as dried residues of ^99^Tc-containing solutions, which could potentially form particles, was avoided or performed under controlled conditions in closed containments with appropriate air exchange.

To obtain the standard, multiple decayed generator eluates were unified to a total amount of ~ 35 mL. ^99^Tc from the combined eluates was cleaned and preconcentrated using TEVA resin. The resin was densely packed into a 500-µL cartridge, preconditioned with HNO_3_ (10 mL, 0.1 M) and loaded with the unified eluates that were spiked with HNO_3_ to a final acid concentration of 0.1 M. After washing with HNO_3_ (10 mL, 1 M), ^99^Tc was eluted in five steps of 100 µL (HNO_3_, 8 M). The first two fractions contained the entire detectable amount of ^99^Tc and were consequently unified and quantified using TXRF analysis.

The quantitative characterization of the cleaned and preconcentrated ^99^Tc standard was carried out with TRXF analysis (S2 PICOFOX spectrometer operated with Bruker Spectra software, version 6.1.5.0; all from Bruker Nano GmbH, Berlin, Germany). The excitation settings for the analysis were set at 50 kV and 750 mA. For quantification, two aliquots of 22.8 µL from the combined TEVA eluates were each mixed with 1.2 µL of a diluted solution of an elemental As standard, so that the final concentration of As in the mixture was 500 µg/L. A volume of 5 µL was pipetted onto two separate quartz disks, dried, and analyzed in triplicate with a recording time of 30 min. Data were smoothened with Savitzky-Golay filtering at a second-order polynomial regression over 15 data points. The emitted signal of Tc was found to be a stack of multiple emission lines of L-alpha and L-beta transitions, which required subsequent deconvolution. Therefore, the most intense signal, originating from the L-alpha 1 emission was isolated by fitting its left flank, unaffected by any overlap, with a gaussian function. The resulting gaussian-shaped curves obtained from the X-ray fluorescence emission of Tc and As were integrated and enabled an accurate calculation of the Tc content in the combined eluates, without the need for a certified Tc reference material. The specific relative sensitivities for Tc and As (${S}_{{\text{Tc}}}$ and ${S}_{{\text{As}}}$), necessary for calculating the concentration of Tc (${c}_{{\text{Tc}}}$), were taken from the instrument software. The concentration of Tc was calculated using Eq. 1, which additionally required the recorded net intensities of Tc and As (${N}_{{\text{Tc}}}$ and ${N}_{{\text{As}}}$) and the concentration of the internal As standard (${c}_{{\text{As}}}$).

1 $${c}_{{\text{Tc}}}={c}_{{\text{As}}}\cdot \frac{{N}_{{\text{Tc}}}/{S}_{{\text{Tc}}}}{{N}_{{\text{As}}}/{S}_{{\text{As}}}}$$

## Results and discussion

### Generation of a ^99^Tc standard

The concept of IBDA developed by Clases et al*.* builds on an IDA-like strategy for internal calibration. Here, a sample containing ^99^Tc is spiked with a Ru solution. With ^99^Ru, Ru possesses an isobaric isotope, which generates a signal that convolutes with the signal of ^99^Tc. The relative contribution of each signal can be deconvoluted when comparing it against the signal of another Ru isotope (e.g., ^101^Ru) and considering relative isotopic abundances of Ru. Subsequently, factoring in element specific sensitivities in ICP-MS allows calibration according to considerations applicable in IDA. This approach has two advantages: First, ^99^Tc can be quantified with a common Ru standard if specific elemental sensitivities are considered. Second, this approach can be used on-line in a post-column setup which allows the calibration of transient signals [10]. Equations 2 and 3 comprise relevant terms for calibration of both total Tc levels (Eq. 2) and transient Tc signals (Eq. 3).

2 $${c}_{{\text{S}}}={c}_{{\text{Sp}}}\cdot \frac{{m}_{{\text{Ru}}}}{{m}_{{\text{Tc}}}}\cdot \frac{{M}_{{\text{Tc}}}}{{M}_{{\text{Ru}}}}\cdot \frac{{A}_{101}^{{\text{Ru}}}}{{A}_{99}^{{\text{Tc}}}}\cdot \left({R'}_{{\text{m}}}-\frac{{A}_{99}^{{\text{Ru}}}}{{A}_{101}^{{\text{Ru}}}}\right)$$

3 $${MF}_{{\text{Tc}},{\text{S}}}={MF}_{{\text{Ru}},{\text{Sp}}}\cdot \frac{{M}_{{\text{Tc}}}}{{M}_{{\text{Ru}}}}\cdot \frac{{A}_{101}^{{\text{Ru}}}}{{A}_{99}^{{\text{Tc}}}}\cdot \left({R'}_{{\text{m}}}-\frac{{A}_{99}^{{\text{Ru}}}}{{A}_{101}^{{\text{Ru}}}}\right)$$

Here, ${c}_{{\text{Tc}},{\text{S}}}$ and ${c}_{{\text{Ru}},{\text{Sp}}}$ represent the concentrations of ^99^Tc in the sample and of Ru in the spike. ${MF}_{{\text{Tc}},{\text{S}}}$ and ${MF}_{{\text{Ru}},{\text{Sp}}}$ correspond to the resulting mass flows of Ru and of ^99^Tc in sample and spike respectively, whereas ${M}_{{\text{Tc}},{\text{Ru}}}$ denote the atomic masses of Tc and Ru. ${m}_{{\text{Ru}},\mathrm{ Tc}}$ is the mass of spike and sample, respectively, and ${R}_{{\text{m}}}^{\mathrm{^{\prime}}}$ is the recorded isotope ratio corrected by mass bias and specific elemental sensitivity of ^99^Ru and ^99^Tc. ${A}_{101}^{{\text{Ru}}}, {A}_{99}^{{\text{Ru}}}$, and ${A}_{99}^{{\text{Tc}}}$ represent the respective natural abundances of ^101^Ru, ^99^Ru, and ^99^Tc. Although ^99^Ru and ^99^Tc are expected to each show comparable specific elemental sensitivity in ICP-MS, small differences in their sensitivities have to be considered as follows [22]. Equation 4 shows how sensitivity correction of the recorded isotope ratio is performed.

4 $${R'}_{{\text{m}}}=\frac{{F}_{{\text{r}}}\cdot \left({{\text{I}}}_{99}^{{\text{m}}}-{{\text{I}}}_{101}^{{\text{m}}}\cdot \frac{{A}_{99}^{{\text{Ru}}}}{{A}_{101}^{{\text{Ru}}}}\right)+\left({{\text{I}}}_{101}^{{\text{m}}}\cdot \frac{{A}_{99}^{{\text{Ru}}}}{{A}_{101}^{{\text{Ru}}}}\right)}{{{\text{I}}}_{101}^{{\text{m}}}}$$

**Fig. 3:**
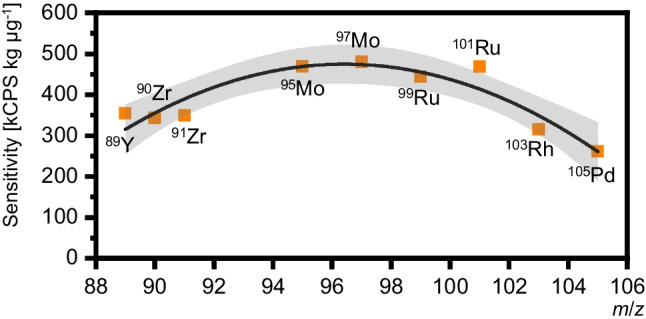
Determination of the specific elemental sensitivity of ^99^Tc by means of ICP-MS via a second-order polynomial fit of surrounding and not overlapping 4d-transition metal isotopes (^89^Y, ^90^Zr, ^91^Zr, ^95^Mo, ^97^Mo, ^99^Ru, ^101^Ru, ^103^Rh, ^105^Pd). Sensitivities were normalized by their natural isotopic abundances. A 95% confidence interval describes the uncertainty of the polynomial fit

**Fig. 4:**
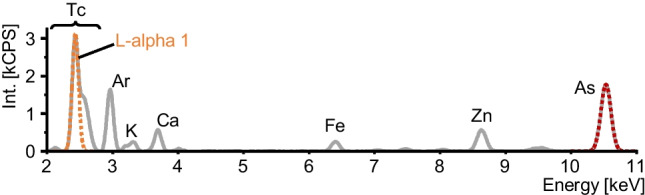
TXRF spectrum obtained for the quantification of the prepared ^99^Tc standard. The gaussian-shaped signals for Tc and the internal standard As were fitted and integrated to calculate the concentration of Tc in each analyzed aliquot using Eq. 1. Remaining residues of the isotonic 0.9% saline solution, not fully separated with TEVA resin, are visible as well. The stacked signal of Tc comprised of multiple emissions of the L-shell transition series was deconvoluted by fitting only the left flank of the main L-alpha 1 emission with a gaussian function. Thereby, a potentially interfering signal originating from chlorine residues at about 2.8 keV was concurrently separated from the signal of Tc

${F}_{{\text{r}}}$ represents the sensitivity correction factor, established as the ratio of the two respective elemental sensitivities of ^99^Ru and ^99^Tc. ${{\text{I}}}^{{\text{m}}}$ denotes the recorded signal on either *m*/*z* 99 or 101. While the sensitivity of ^99^Ru can be determined directly with a commercial certified elemental standard, accessing the sensitivity of ^99^Tc poses much more of a challenge as standards are generally not available. In an earlier approach, Clases et al*.* estimated the requested elemental sensitivity via an interpolation of the surrounding 4d-transition metals as they all show comparatively similar first ionization energies [22]. Yet, this approximation approach holds an intrinsic uncertainty characterized as the width of the 95% confidence band around the polynomial function [10, 22]. The uncertainty for this approach was here estimated to be 10% (compare Fig. 3) and derived from the interpolation between sensitivities of the elements from the 4d-transition metal series. The uncertainty was strongly depending on the instrumental setup and tuning parameters. To improve the detection power for the target element Tc and Ru as calibrant, instrument performance was optimized by considering *S*/*N*. Consequently, for a more accurate determination of the ^99^Tc concentration, a direct approach for correcting differences in elemental sensitivity was favorable. This was achieved by obtaining ^99^Tc through a chromatographic generator used in clinical settings. Using extraction chromatography with TEVA resin, ^99^Tc was purified and preconcentrated from multiple combined raw ^99m^Tc-generator eluates until concentrations reached the linear dynamic range of TXRF analysis, which for Tc was located above 1 mg L^−1^ [28]. X-ray fluorescence analysis allowed the quantification of ^99^Tc in the TEVA eluate while using an internal As standard. Two aliquots of the combined TEVA eluates were each analyzed in triplicate to determine the newly generated ^99^Tc standard at a concentration of 36.1 ± 0.2 mg L^−1^. This subsequently allowed direct determination of the specific elemental sensitivity of ^99^Tc in ICP-MS for the on-line application of IBDA (Eq. 3). An exemplary TXRF spectrum is presented in Fig. 4.

**Fig. 5:**
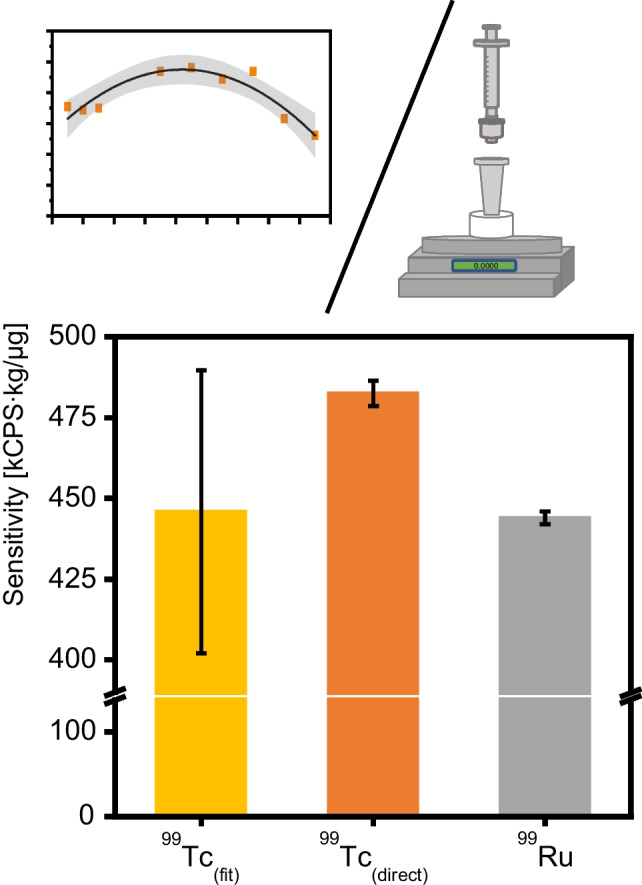
Comparison of the determined specific elemental sensitivities of ^99^Tc determined via approximation based on neighboring 4d-transition metals, of ^99^Tc determined directly from a generated standard and of ^99^Ru in ICP-MS

Figure 5 compares sensitivities determined using a ^99^Tc standard with the sensitivity approximation method previously described. It is visible that the sensitivity estimated via the approximation approach had a higher uncertainty. While the sensitivity determined via the manufactured ^99^Tc standard still lay within the confidence interval of the previous method, the prepared standard significantly improved uncertainty to only 0.8%.

### Anion-exchange chromatography of [^99^Tc]Tc${\text{O}}_{4}^{-}$

**Fig. 6:**
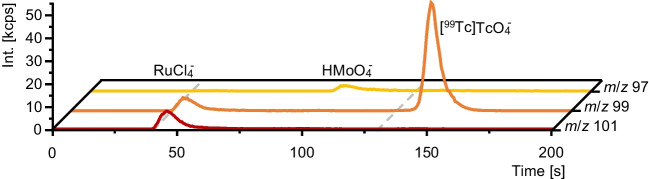
Chromatograms of the monitored *m*/*z* from Mo, Ru, and Tc for the developed separation of [^99^Tc]Tc${\text{O}}_{4}^{-}$ (all 1 µg L^−1^). Dashed lines indicate the switching points of the three-step gradient at 40 s and 130 s. Mass traces were not normalized for their natural isotopic abundances to allow better comparison between individual signals and noise levels

In aqueous samples and under aerobic conditions, ^99^Tc is expected to occur as stable [^99^Tc]Tc${\text{O}}_{4}^{-}$, which due to its high polarizability requires strong elution strength on an IC column. To enable fast and robust speciation analysis of [^99^Tc]Tc${\text{O}}_{4}^{-}$ in aqueous samples, separation was optimized to avoid coelution of the two potentially interfering species. For method development, a [^99^Tc]Tc${\text{O}}_{4}^{-}$ sample was spiked with ${\text{MoO}}_{4}^{2-}$ and ${\text{RuCl}}_{4}^{-}$. With the developed method, [^99^Tc]Tc${\text{O}}_{4}^{-}$ was well separated from both interferences in less than 3 min (Fig. 6).

To evaluate the chromatographic method and the quantification of [^99^Tc]Tc${\text{O}}_{4}^{-}$ by means of IBDA while using a system with ion suppression and aerosol desolvation, recoveries were determined (Table 1): The recoveries of the chromatographic column and of the suppressor were determined individually with injections of the in-house generated elemental ^99^Tc standard. The chromatographic column and suppressor showed only minor influences on recovery of ^99^Tc indicating quantitative elution of Tc${\text{O}}_{4}^{-}$. At low ^99^Tc concentrations, recoveries decreased slightly but were still well above 90%.

**Table 1:** Recoveries for [^99^Tc]Tc${\text{O}}_{4}^{-}$ were determined for the IC column and suppressor individually. For the IC column, different concentrations were tested. All errors refer to calculated standard deviations from the triplicate analysis

Method segment	c(^99^Tc) in ng L^−1^	Recovery in %
IC column	10	94 ± 3
	50	97 ± 2
	100	102 ± 1
	500	103 ± 2
	1000	101 ± 1
Suppressor	100	94 ± 2

To determine the accuracy of on-line IBDA, a known amount of ^99^Tc obtained from the in-house quantified standard was analyzed. Levels determined by on-line IBDA and TXRF deviated only by 1%. Additionally, the robustness and repeatability of the chromatographic separation were investigated in the presence of several other elements from the 4d-transition metal series (Y, Zr, Nb, Mo, Ru, Rh, Pd, and Cd; all *c* = 1 µg/kg), which may act as interferences (Table 2). ^99^Tc was quantified in an eluate sample obtained from a ^99m^Tc-generator with and without spiking different 4d-transition metals as simulated contamination. Table 2 compares the levels of ^99^Tc determined by on-line IBDA with and without the presence of potentially confounding 4d elements as well as determined via off-line IBDA (Eq. 2). Determined values only deviated slightly indicating that on-line IBDA enabled robust quantification. The error derived from these analyses given as the standard deviation of injecting triplicates, was calculated to be between 2.6% (with spiked 4d-element contamination) and 3.6% (pure eluate). The general uncertainty of the presented IC-ICP-MS method was estimated to be not more than 4%. Limits of detection (LOD) and quantification (LOQ) were estimated by considering the standard deviation of the signal of the internal Ru standard multiplied with 3 or 10, respectively, and dividing it by the specific elemental sensitivity of ^99^Tc. Following this approach, LOD and LOQ were calculated to be 0.67 ± 0.04 ng kg^−1^ and 2.2 ± 0.1 ng kg^−1^, respectively.

**Table 2:** Comparison of manually performed off-line IBDA with automated on-line IBDA of either a pure or a 4d-transition metal contamination-spiked ^99m^Tc-generator eluate sample. All errors refer to calculated standard deviations from the triplicate analysis

	Off-line IBDA	On-line IBDA	On-line IBDA (4d-spike)
c(Tc)_raw eluate_ (µg L^**−**1^)	443 ± 2	422 ± 15	421 ± 11
Ratio on-line/off-line in %	-	95 ± 4	95 ± 3

### Quantification of [^99^Tc]Tc${\text{O}}_{4}^{-}$ in urine

**Fig. 7:**
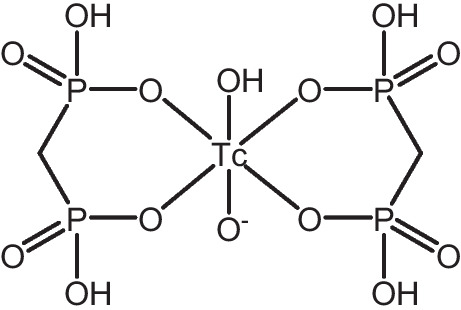
One suggested structure of [^99m^Tc]Tc-MDP [13, 33]

The developed method has the capability to determine pertechnetate in complex samples and may have a utility to trace it in environmental or (bio-)medical scenarios. In a proof of principle, a urine sample obtained from a patient, who on the same day underwent bone scintigraphy with [^99m^Tc]Tc-MDP (Fig. 7), was analyzed. Following filtration, 50 µL of raw and undiluted urine was directly injected onto the IC column according to the schematic setup presented in the experimental section (Fig. 2). Consequently, the overall preparation effort for the sample was low and took only about 2 min of filtration time. The urine sample was chosen as complex specimen containing only trace amounts of Tc to showcase the capabilities of the developed method.

**Fig. 8:**
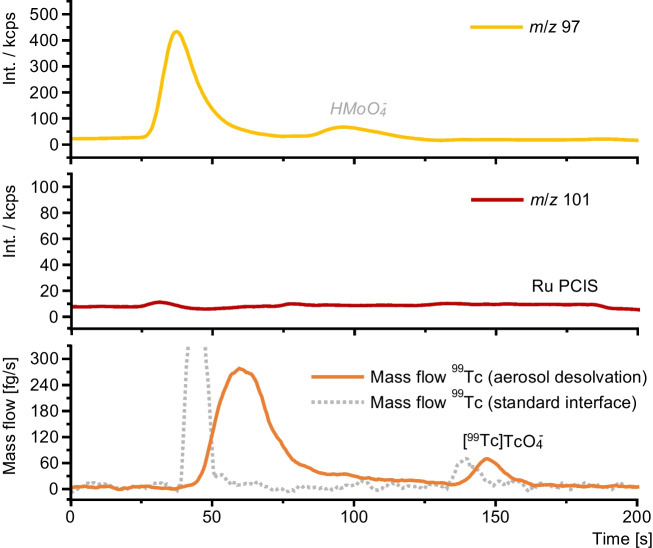
Chromatographic separation of a urine sample from a patient having undergone bone scintigraphy with [^99m^Tc]Tc-MDP on the same day. Mass traces of ^97^Mo as a major contaminant and ^101^Ru as a potential contaminant and as the added PCIS are shown. ^99^Tc is shown as the calculated and corrected mass flow obtained using Eq. 3. For comparison with the setup using aerosol desolvation, the mass flow obtained from the standard setup was indicated with dashed lines

Chromatograms of Mo and Ru as potential contaminants as well as the added PCIS were recorded (Fig. 8). Here, the corrected mass flow of ^99^Tc was calculated and plotted using Eq. 3. For comparison with the setup using aerosol desolvation, the mass flow obtained from the standard setup with a double pass spray chamber was indicated with dashed lines. Mo, most likely originating from endogenous sources, could be separated from the expected retention time window of [^99^Tc]Tc${\text{O}}_{4}^{-}$ at 145 s. Ru could not be detected in the urine sample. The two mass flows of ^99^Tc, obtained with the used aerosol desolvation setup or the compared standard setup, showed some differences in peak shape, retention times, and noise level. Although the peaks expectedly appeared broader when using the suppressor and the aerosol desolvation system, the main advantage of the developed setup became apparent: As the signal of the baseline was significantly more stable due to generally higher recorded intensities, trace level determinations of lower signals like the peak of [^99^Tc]Tc${\text{O}}_{4}^{-}$ could still be performed (*S*/*N* = 68), which was not the case when using a conventional nebulization setup.

In previous studies tackling the impurities of synthesized Tc-based tracers, we could demonstrate that tracers show residues of free [^99^Tc]Tc${\text{O}}_{4}^{-}$, which either were not converted to the tracer or were generated due to degradation [10]. Data from previous publications indicated a high stability of the formed [^99m^Tc]Tc-MDP tracer maintaining labelling yields of over 95% even several hours after formulation when kept in the labelling medium [29]. Nevertheless, in addition to residues from the labelling process, [^99^Tc]Tc${\text{O}}_{4}^{-}$ could form through oxidation in metabolic processes. In this study, a concentration of 19.6 ± 0.5 ng L^−1^ was determined by integrating the calculated mass flow of ^99^Tc at the retention time window of [^99^Tc]Tc${\text{O}}_{4}^{-}$. Quantitative data on the residue of [^99^Tc]Tc${\text{O}}_{4}^{-}$ in urine obtained via the presented method could therefore help to further understand pharmacokinetic parameters of tracers.

In the mass flow of ^99^Tc, a second peak could be observed at shorter retention times of approximately 50 s (Fig. 8) and was likely the intact tracer, which was quantified at 149 ± 4 ng L^−1^. The determined error of both recorded peaks, represented by the standard deviation of analyzing triplicates ([^99^Tc]Tc${\text{O}}_{4}^{-}$, 2.5%; [^99^Tc]Tc-MDP, 2.7%), was in good accordance with the overall uncertainty of the method, determined earlier to be less than 4%. Due to this comparably low concentration, the non-volatile buffer used for the separation, and the strong ionic matrix of the urine sample, additional studies using molecular mass spectrometry to further investigate the structure of the compound, similar to previous studies performed with pure tracers, could not be performed [10]. However, the found concentration matches typically administered tracer dosages for bone scintigraphy with [^99m^Tc]Tc-MDP, which depend on the weight of the patient and sometimes also the expected malignancy of the tumors. Typical dosages for adult patients may reach up to 13 MBq kg^−1^ bodyweight equaling injected amounts of 67 pg ^99m^Tc per kg bodyweight [8, 30]. The expected concentrations in urine eventually vary with the amount of urine produced after the examination and with the overall renal function [31, 32].

## Conclusions

In this work, a new method employing anion-exchange chromatography was developed and coupled to inductively coupled plasma–mass spectrometry (ICP-MS). This setup was used to quantify [^99^Tc]Tc${\text{O}}_{4}^{-}$ in urine obtained from a patient after scintigraphic examination. For this purpose, an on-line calibration approach (IBDA) was used to determine transient Tc signals. For accurate quantification in ICP-MS, IBDA required knowledge of the specific elemental sensitivities. Therefore, an in-house generated and counter-quantified elemental ^99^Tc standard was prepared enabling significantly higher accuracy and reduced uncertainty compared to previous approaches. Generating a standard from decayed medical ^99m^Tc-generator eluate by means of extraction chromatography and counter-quantification by means of total reflection X-ray fluorescence (TXRF) analysis has proven to be an effective way of obtaining ^99^Tc for any referencing purposes in future applications, as availability of certified Tc standards still is lacking.

The detection power of the presented method for ^99^Tc could be further improved by applying aerosol desolvation nebulization to ICP-MS, which increased aerosol transport efficiency. LOD and LOQ were calculated to be 0.67 ± 0.04 ng kg^−1^ and 2.2 ± 0.1 ng kg^−1^, respectively, reaffirming the enhanced detection capabilities provided by the combination of aerosol desolvation and the IC-ICP-MS setup. Its use for desolvation of non-volatile eluents was enabled by the introduction of a cation suppressor, preventing crystal formation inside the desolvation unit. Thereby, the range of strong matrices or non-volatile eluents was made accessible for future chromatographic applications of aerosol desolvation nebulization, which before exclusively required entirely volatile buffers.

In a proof of principle, the method was used to determine ^99^Tc species in undiluted urine. [^99^Tc]Tc${\text{O}}_{4}^{-}$ (c(^99^Tc) = 19.6 ± 0.5 ng L^−1^) and another ^99^Tc-containing species (c(^99^Tc) = 149 ± 4 ng L^−1^) were determined and the latter species was most likely the injected [^99m^Tc]Tc-MDP tracer used for bone scintigraphy.
